# The challenges of the genome-based identification of antifungal resistance in the clinical routine

**DOI:** 10.3389/fmicb.2023.1134755

**Published:** 2023-04-20

**Authors:** Ana Alastruey-Izquierdo, Antonio J. Martín-Galiano

**Affiliations:** ^1^Mycology Reference Laboratory, National Centre for Microbiology, Instituto de Salud Carlos III, Madrid, Spain; ^2^Center for Biomedical Research in Network in Infectious Diseases (CIBERINFEC-CB21/13/00105), Instituto de Salud Carlos III, Madrid, Spain; ^3^Core Scientific and Technical Units, Instituto de Salud Carlos III, Madrid, Spain

**Keywords:** amphotericin B, antimicrobial target, *Aspergillus*, azoles, *Candida*, *Cryptococcus*, echinocandins, polyenes

## Abstract

The increasing number of chronic and life-threatening infections caused by antimicrobial resistant fungal isolates is of critical concern. Low DNA sequencing cost may facilitate the identification of the genomic profile leading to resistance, the resistome, to rationally optimize the design of antifungal therapies. However, compared to bacteria, initiatives for resistome detection in eukaryotic pathogens are underdeveloped. Firstly, reported mutations in antifungal targets leading to reduced susceptibility must be extensively collected from the literature to generate comprehensive databases. This information should be complemented with specific laboratory screenings to detect the highest number possible of relevant genetic changes in primary targets and associations between resistance and other genomic markers. Strikingly, some drug resistant strains experience high-level genetic changes such as ploidy variation as much as duplications and reorganizations of specific chromosomes. Such variations involve allelic dominance, gene dosage increments and target expression regime effects that should be explicitly parameterized in antifungal resistome prediction algorithms. Clinical data indicate that predictors need to consider the precise pathogen species and drug levels of detail, instead of just genus and drug class. The concomitant needs for mutation accuracy and assembly quality assurance suggest hybrid sequencing approaches involving third-generation methods will be utilized. Moreover, fatal fast infections, like fungemia and meningitis, will further require both sequencing and analysis facilities are available in-house. Altogether, the complex nature of antifungal resistance demands extensive sequencing, data acquisition and processing, bioinformatic analysis pipelines, and standard protocols to be accomplished prior to genome-based protocols are applied in the clinical setting.

## 1. Introduction

Genome-based predictors of antimicrobial resistance (AMR) are not available for fungal pathogens. In contrast, the extensive knowledge of resistance markers is widely applied in bacterial isolates for the automated identification of the AMR genome section, the resistome. The performance accuracy of some of these methods equals antibiograms, the current standard in routine laboratories. Among AMR bacteria, the six ESKAPE organisms cause most intra-hospital cases (Rice, 2008). Resistome identification helps to rationally guide the treatment to prevent therapeutic failure and raise of future resistance levels in the principal bacterial pathogens.

Antifungal resistance arises by selective pressure after long treatment and/or wide utilization of antifungal clinical agents (Cowen, 2008) besides fungicides with agricultural purposes (Snelders et al., 2012). Prophylaxis is compromised by the several hurdles that prevent the development of vaccines to protect against fungal diseases (Oliveira et al., 2021). Therefore, despite resistance, control of fungal infections still exclusively relies on antimicrobial therapy. More than 95% clinical cases, including those refractory to treatment, are caused by three genera: *Cryptococcus*, *Candida,* and *Aspergillus* (Brown et al., 2012).

Several protocols have approached the bacterial resistome with different degrees of drug and pathogen inclusivity (Scaria et al., 2005; Liu and Pop, 2009; Gupta et al., 2014; de Man and Limbago, 2016; Alcock et al., 2020; Bortolaia et al., 2020). They commonly consist on a database of resistance determinants, a detection algorithm, and a controlled vocabulary scheme that connects the whole body of information to produce a readable outcome. Resistance markers are either the presence of genes (the “gene mode”), e.g., the *mecA* gene in beta-lactam-resistant *Staphylococcus aureus*, or specific polymorphisms in core genes (the “variant mode”), e.g., the S87W mutation in DNA Topoisomerase IV subunit A of fluoroquinolone-resistant *Pseudomonas aeruginosa*. However, higher biological complexity, multichromosomal organization, and lower omic data availability complicate the development of equivalent tools for eukaryotic pathogens as fungi or parasites (Leprohon et al., 2015).

Given the unquestionable interest on fungal resistome protocols, we objectively discuss here whether the construction of such tools is appropriate and realistic, and which aspects should be improved prior to meet their goals.

## 2. Perspective on antifungal resistome predictors

### 2.1. Is antifungal resistance relevant enough to justify genome-level approaches?

Resistome predictors are laborious initiatives only justified when provide evident clinical benefits. AMR bacteria show high virulence and national cost burden, associated with 5 million deaths and 1.3 direct causality in 2019 (Antimicrobial Resistance Collaborators, 2022). In 2017, the World Health Organization (WHO) presented a list with the top-12 bacterial pathogens (Tacconelli et al., 2018), which are extensively covered by resistome tools.

Fungal infections amount to 1.6 million deaths in developing countries (Almeida et al., 2019). *Cryptococcus neoformans, Candida auris, Candida albicans,* and *Aspergillus fumigatus* conform the critical priority group in the recently released Fungal Priority Pathogen List by the WHO.^1^ An increment of candidiasis caused by different *Candida* species has been observed (Lamoth et al., 2018), particularly *C. auris* in Europe (Kohlenberg et al., 2022). Fungal pathogens are gaining relevance in risk groups due to aging, AIDS, cancer chemotherapy, organ-transplanted patients, viral infections (flu and COVID-19), or ICU admission. Some cases can be deadly, such as systemic fungemia, pneumonia, and meningitis if not treated timely and properly. *Candida* is the fourth pathogen more often found in sepsis in the United States (Delaloye and Calandra, 2014).

**Figure 1:**
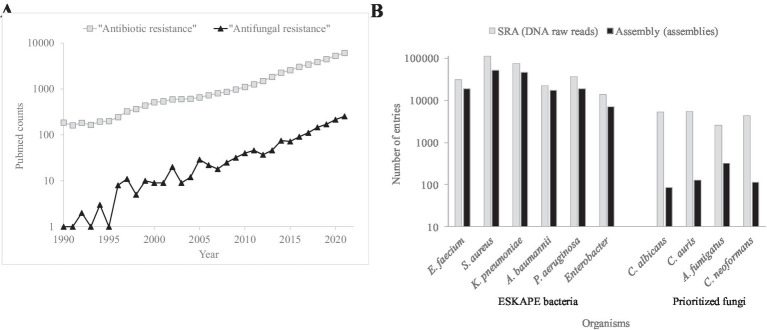
Occurrence of literature entries and number of genomes for AMR bacteria and fungi. **(A)** Number of PubMed (https://pubmed.ncbi.nlm.nih.gov/) counts during the 1990–2021 period using the “Antibiotic resistance” and “Antifungal resistance” search terms. **(B)** Number of entries concerning DNA reads and assembled genomes for the principal AMR bacteria and fungi in the SRA (https://www.ncbi.nlm.nih.gov/sra) and Assembly (https://www.ncbi.nlm.nih.gov/assembly) NCBI databases, respectively.

Mortality and morbidity associated to AMR fungi is increasing (Perlin et al., 2017; Fisher et al., 2022; Gow et al., 2022; Figure 1A). The antimicrobial control of these infections is compromised by the small drug arsenal and genomic plasticity. Fungi are eukaryotic, i.e., molecularly similar to humans, which limits the number of antifungal family drugs to five. Only azoles, echinocandins, and polyenes are used to treat systemic infections. Genome plasticity facilitates the adaptive resistance to chemotherapeutical options during long treatments. Multidrug resistant phenotypes are also detected (Pfaller et al., 2012; Pham et al., 2014). In contrast to adaptive resistance, intrinsic resistance, caused by naturally occurring ancestral mutations, has also been detected (Chowdhary et al., 2018). Especially worrisome are the intrinsic pan-resistance observed in *Lomentospora prolificans* (Wu et al., 2020) and the emergence of fluconazole resistant *Candida parapsilosis* besides its intrinsic higher MICs to echinocandins (Trevijano-Contador et al., 2022). The therapeutic failure using the two preferred drug classes forces the prescription of alternative treatments such as amphotericin B, the antifungal with a broadest spectrum.

### 2.2. Would resistome predictors improve simpler molecular methods?

Fungal infections are usually treated by empirical therapy based on historical records or after applying phenotypical methods. Antifungal susceptibility testing involves the standardized exposition of the fungus to different concentrations of drug in liquid or solid media using reference protocols (CLSI and EUCAST). Therapeutic efficacy can be therefore predicted by quantitatively assessing the minimum inhibitory or effective concentrations. Clinical break points for some antifungals and species are available. Disadvantages of phenotypical methods include low reproducibility by subtle operational changes and potential absence of cutoff points. Besides, they are labor-intensive and time-consuming which has a direct impact in mortality (Garey et al., 2006; Chamilos et al., 2008).

Alternatively, the identification of mutations by sequencing outperforms MIC for antifungal therapy success (Shields et al., 2012; Alexander et al., 2013). However, only a limited number of genes can be sequenced by conventional Sanger sequencing or screened by real-time PCR (Durand et al., 2021). Resistance marker expression can be approached by MS MALDI-TOF (Vella et al., 2017; Maenchantrarath et al., 2022), which shows short turn-around-times but lacks of standards for universal application (Durand et al., 2021).

The polygenic nature of antifungal resistance (Gonçalves et al., 2016) favors the utilization of massive techniques. Cost-effective new generation sequencing (NGS) alone or complemented with better genome assemblies achieved by third-generation sequencing (TGS) facilitates obtaining whole-genome sequences. Genomics provides a view of the strain genetics at the highest, nucleotide-level, resolution, and the simultaneous evaluation of a multiplicity of factors (Garnaud et al., 2015; McTaggart et al., 2020; Schikora-Tamarit and Gabaldón, 2022b).

### 2.3. How many antifungal resistance determinants are precisely described?

A central question is whether the current scientific knowledge includes enough fungal AMR mechanisms to fuel the development of efficient resistome predictors. Laboratory and clinical studies have indeed described numerous adaptive and intrinsic antifungal AMR processes with molecular precision.

Many point mutations linked to AMR have been characterized, which would be akin to the “variant mode” of bacterial resistome predictors. These include those in primary antifungal targets: the key enzyme for ergosterol biosynthesis sterol 14α demethylase for azoles, and glucan synthase for echinocandins. Many of these mutations produce residue changes in relevant (hot-spot) zones like drug binding pockets (Garcia-Effron et al., 2009). Mutations in non-primary target proteins can also produce resistance, such as in the ergosterol biosynthesis pathway to azoles (Alcazar-Fuoli and Mellado, 2012). Loss-of-function (LOF) resistance mutations, such as premature stop codons and insertion of transposable elements, have been described for instance in the ergosterol metabolism enzyme ERG3 (Lupetti et al., 2002; Ksiezopolska et al., 2021). Intrinsic AMR examples are the numerous polymorphisms in ERG11 and FKS proteins, causing azole and echinocandin resistance in *C. auris* (Muñoz et al., 2018) and *C. parapsilosis* (Garcia-Effron et al., 2008), respectively. Decrease of the intracellular drug concentration to ineffective levels can be achieved by gain-of-function (GOF) mutations in positive regulators of antifungal efflux pumps (Cannon et al., 2009). AMR phenotypes are favored in isolates with hypermutator behavior due to altered DNA repair systems (Healey et al., 2016). Resistant phenotypes caused by point mutations are usually only observed in diploid fungi when the sensitive allele is lost (Garnaud et al., 2015).

Expression-driven resistance can also be obtained by: (i) mutations in response elements of *cis* promoters of efflux pumps (Gaur et al., 2004); (ii) new promoters introduced by transposable elements (Hu et al., 2021); (iii) longer mRNA stability by enzymatic variants that increase polyadenylation (Manoharlal et al., 2010); and (iv) LOF mutations of lysine-acetylation enzymes of histones *via* epigenetic chromatin modification (Orta-Zavalza et al., 2013; Tscherner et al., 2015).

Fungal AMR often involve large chromosomal changes affecting copy number and expression regime of genes. Large changes include ploidy alterations, the combination of chromosomes into new ones, and the generation of isochromosomes (Selmecki et al., 2006, 2008; McTaggart et al., 2020). For example, chromosomal monosomies have been associated with tolerance to fluconazole and 5-flourocytosine (Yang et al., 2013; Uddin et al., 2021). The copy number of genes coding primary targets can be also increased by local duplication (Chow et al., 2020).

### 2.4. Are known antifungal resistance determinants identifiable through genome sequencing?

An essential issue for routine antifungal resistome is how many fungal AMR markers are detectable by WGS and with which degree of automation.

Intrinsic resistance involves precise taxon identification. For that, k-mers thresholds for species assignation have been reported (Gostinčar, 2020). Precise sub-species detection requires the application of phylogenomic tools or the standard sequence type, ST, scheme (Taylor and Fisher, 2003).

Point polymorphisms associated to AMR follow the classical “variant-mode” resistome principles. Illumina NGS technology meets quality and coverage standards enough to identify LOF and GOF residue changes, premature stop codons, small indels, and gene duplications (Garnaud et al., 2015). Protocols such as OVarFlow and SnpEff are available to call and study point and small indels in eukaryotic genomes (Bathke and Lühken, 2021; Cingolani, 2022). Fungal-specific and other tools have been already designed for this task, such as YMAP (Abbey et al., 2014). Deep coverages are also adequate to find heteroresistant subpopulations (Zhai et al., 2022). Homozygosity (~100% reads) and heterozygosity (~50% reads) are approachable using the proportion of mutated reads (Spettel et al., 2019).

Large chromosomal changes are harder to parametrize although they can be evaluated through tools like PerSVade (Schikora-Tamarit and Gabaldón, 2022a) or Assemblytics (Nattestad and Schatz, 2016). NGS permits proportional read count alteration in certain chromosomes or in the vicinity context of marker genes. Transposable elements are identifiable using sequence profiles from databases like Repbase (Kapitonov and Jurka, 2008). However, genome environment modifications through transposons and chromosomal rearrangements may require high-quality assemblies as those provided by TGS platforms.

The nature of some antifungal resistance modes is still too opaque to be investigated. The resulting alteration profiles by genomics can be a mixture of AMR causal, linked, suspected, or irrelevant changes. Players include drug targets, regulators, pathways, promoters, mRNA polyadenylation, and lysine acetylases. To assess the relevance of sequence variations, the number of sequenced genomes and the molecular knowledge of fungal pathogens should be strengthened (Figure 1B).

### 2.5. How practical is genome sequencing in the clinical setting?

Beyond performance in the research environment, the ultimate goal of antifungal resistome predictors is their clinical application. Full advantage of genome-based tools has been taken for bacterial pathogens in the clinical laboratory in the recent years (Deurenberg et al., 2017).

Fungal resistome tools should be economically efficient, in particular in developing countries. The number of samples per NGS run should be optimized. The number of reads and assemblies obtained by NGS should satisfy quality metrics such as high genome coverage and a small contig number. Concerning TGS, nanopore flongle cells or multiplexing show lower fidelity but are cost-effective options for NGS-TGS hybrid approaches (Lipworth et al., 2020) or utilization in low- and middle-income countries. Expense can also be reduced by targeted resequencing (Spettel et al., 2019).

A principal hurdle in genomics is the downtime to obtain sequencing results. Long turnaround-times may be assumable for chronic non-critical superficial infections (like candiduria), but not for life-threatening ones (like fungemia, pneumonia or meningitis) that could be mortal within 24 h. The later demands in-house facilities as dependence of third externalized parties can substantially delay outcomes (Raven et al., 2018). Thus, the initial investment to purchase NGS facilities would be eventually profitable. Furthermore, nanopore technologies are relatively cheap and can be escalated up to 48 simultaneous cells.

The requirement of a high bioinformatic knowledge may also hamper the predictor utilization. Unexperienced laboratory staff should be trained to execute and interpret the provided software. On-line resources that provide human readable reports, such as Pathogenwatch^2^ and Microreact (Argimón et al., 2016), besides handy computational environments like Galaxy (Galaxy Community, 2022) may facilitate analyses for laboratories worldwide.

## 3. Discussion

The wide application of genome-based therapy is still far for fungal infections. Worrying epidemiological data support the recent decision by the WHO of raising prioritization to control difficult-to-treat fungal pathogens, particularly opportunistic ones in immunocompromised patients. The reduced number of licensed antifungals highlights the relevance to discern susceptible phenotypes to prevent exhaustion of the therapeutic options. Accurate antifungal susceptibility methods are needed (Pristov and Ghannoum, 2019), which allows for the application of rational antifungal regimes. Over empirical multiple therapy, this may reduce long hospital admission timeframe, sequelae, death, high treatment cost, and future resistance (Cowen et al., 2014). Several antifungal resistance modes have been described at molecular detail and algorithms to potentially detect them are accessible. However, some intermediate milestones to develop resistome predictors are not straightforwardly achievable as fungal AMR present additional complex mechanisms respect to bacteria (Table 1). Here, we have objectively evaluated which specific steps are presently solved and which are pending prior to have equivalent tools to bacterial resistome predictors.

**Table 1:** Antimicrobial resistance issues for bacterial and fungal pathogens.

Issue	Bacteria	Fungi
Mutations in targets	+	+
Mutations in regulators	+	+
Efflux pumps	+	+
Mobile genetic elements	Insertion sequences, transposons, phages, plasmids	Transposons
Promoter regions	+	+
Heteroresistance	+	+
Allelic dominance	−	+
Chromosomal aploidies	−	+
Isochromosomes	−	+
Long chromosomal recombinations	−	+
Epigenetic changes	Unknown	+

A strong limitation in the field is the incomplete understanding of some antifungal resistance modes. Fungal molecular biology is intricate and omic data is scarce. Moreover, fundamental knowledge of genetic mechanisms involved in resistance is currently biased toward *C. albicans*, which is not supported by recent epidemiological changes observed in this genus (Logan et al., 2020). Other primary pathogens, such as *A. fumigatus* (Bueid et al., 2010), remain comparatively neglected. Constraints also include polygenic resistant phenotypes, resulting from the confluence of several distributed markers, and the potential absence of a consensus reference strains. The completion in resistance modes, drugs, and species in cost- and time-efficient manner may benefit from multi-disciplinary consortia organized by the mycology community. Increases in the number of sequenced genomes, from multiple platforms, will permit to know the basal sequence variability, reveal previously unknown resistance determinants, precise typing, and virulence assessment. It should be noted that the contribution of databases to fungal resistome predictors ultimately depends on their quality. Likewise, experimental screenings showed a range of success in finding novel resistance modes (Snelders et al., 2008; Van Rhijn et al., 2021; Rhodes et al., 2022).

Compared to simpler monochromosomal bacteria, antifungal resistome software demands a substantial programming upgrade to cover a wide array of genetic events. Point residue mutations or premature stop codons just require simple gene detection and alignment. However, fungal AMR commonly involves gross chromosomal changes and multistep functional pathways. Algorithms to handle some of these issues are available but should be adapted to antifungal antecedents prior to their inclusion in pipelines. The software should operate on associated curated resources as databases that incorporate all reported advances on a regular basis. Such efforts require dedicated human staff and stable financing to prevent discontinuation. Pipelines should satisfy high standards prior approval for clinical decision-making in real patients.

Ideally, fungal resistome predictors would be fueled by the tripartite combination of raw reads, assemblies, and long-read approaches. The availability of in-house sequencing facilities and specialized bioinformatic staff will widen even more the differences also in time-turnaround terms. Consequentially, the sequencing costs and immediacy associated with fulminant infections will be prohibitive for tight budgets. We therefore envisage the screening of all resistance modes in routine will be progressively and unevenly, rather than universally, incorporated into healthcare centers.

Data sharing is facilitated by the standardization of genomic information. Cutoffs for sequencing and assembly quality metrics should be optimized for reproducible protocols that meet clinical-level criteria. The integration of data of different natures into a single system demands the development of a controlled language similar in spirit to the Antibiotic Resistance Ontology (Alcock et al., 2022). Such framework for terms and their connections must capture the correct hierarchical and granular nature of fungal AMR. For instance, the G54E mutation in CYP51A protects *A. fumigatus* against itraconazole, but not to voriconazole among the azole class (Leonardelli et al., 2017). Likewise, resistance can pertain to genus, species, or even sub-species, as the intrinsic azole resistant clade A observed in *A. fumigatus* (Rhodes et al., 2022). Markers should be causally associated to resistance through information about the protein involved, type of mutation (point, stop codon, and deletion), sequence position, location in hotspots, the exact resistance change observed, the GOF or LOF regime to exhibit resistance, and whether the resistant allele is phenotypically dominant or not. Taxon assignation plays a central role to infer the ploidy level and, subsequently, allelic dominance concerns, e.g., haploid for *C. glabrata*. For large changes, genetic environment descriptors of markers such as their chromosomal positioning and flanking genes should be considered. For regulatory issues, the promoter composition (integrity of responsive boxes) and the identification of adjacent transposable elements (type, orientation and integration sites) must be defined. Potential hypermutator behavior, heteroresistance indicators, and epigenetic changes should also be recorded. The degree of resistance may be binned into categories where high-level resistance may be achieved by the synergy of progressive multiple “intermediate” mechanism (Sasse et al., 2012) or by an unique event (Ferrari et al., 2009). Resistance determinants must be supported by literature identifiers, such as PMID or DOI. Clinical resistome initiatives will thus require strong data normalization and integration efforts.

Based on the above, the steps of the roadmap to follow recurrently may be: (i) exhaustive literature search for described resistance determinants where web-based specific and broad-purpose resources such as FunResDb (Weber et al., 2018) and MARDy (Nash et al., 2018) provide excellent starting material; (ii) conduction of extensive laboratory screenings (Dunyach et al., 2011; Rivero-Menendez et al., 2019) to map the mutational space leading to resistance (Schikora-Tamarit and Gabaldón, 2022b); (iii) generation of updated databases; and (iv) building resistome prediction pipelines.

Despite difficulty, the current status is, in our opinion, mature enough to warrant the design of fungal resistome pioneer tools. The first-generation algorithms should cover current data of *Aspergillus*, *Candida,* and *Cryptococcus*, and be further refined by more species, resistance modes, and quality criteria. Solving pending issues will be presumably cumbersome and demand a strong effort of the scientific community. Nevertheless, it will provide unprecedented performance for routine clinical management and surveillance of these challenging infections.

## Data availability statement

The original contributions presented in the study are included in the article, further inquiries can be directed to the corresponding authors.

## Author contributions

AA-I and AM-G conceived the study, drafted the manuscript, revised the final version of the manuscript, and granted funding. All authors contributed to the article and approved the submitted version.

## Funding

This study was supported by the Acción Estratégica en Salud from Fondo de Investigaciones Sanitarias, ISCIII, grants MPY 509/19 and PI20CIII/00043.

## Conflict of interest

AA-I has received honoraria for educational talks of behalf of Gilead and Pfizer, outside of the submitted work.

The remaining author declares that the research was conducted in the absence of any commercial or financial relationships that could be construed as a potential conflict of interest.

## Publisher’s note

All claims expressed in this article are solely those of the authors and do not necessarily represent those of their affiliated organizations, or those of the publisher, the editors and the reviewers. Any product that may be evaluated in this article, or claim that may be made by its manufacturer, is not guaranteed or endorsed by the publisher.

## Footnotes

^1^ https://www.who.int/publications/i/item/9789240060241

^2^ https://pathogen.watch/
